# Rationale and design of IMPACT-women: a randomised controlled trial of the effect of time-restricted eating, healthy eating and reduced sedentary behaviour on metabolic health during chemotherapy for early-stage breast cancer

**DOI:** 10.1017/S0007114522003816

**Published:** 2023-09-14

**Authors:** Rebecca A. G. Christensen, Mark J. Haykowsky, Michelle Nadler, Carla M. Prado, Stephanie D. Small, Julia N. Rickard, Edith Pituskin, D. Ian Paterson, John R. Mackey, Richard B. Thompson, Amy Ashley Kirkham

**Affiliations:** 1 Faculty of Kinesiology & Physical Education, University of Toronto, Toronto, ON, Canada; 2 Faculty of Nursing, University of Alberta, Edmonton, AB, Canada; 3 Department of Medical Oncology and Hematology, Princess Margaret Cancer Centre, Toronto, ON, Canada; 4 Department of Agriculture, Food and Nutrition Science, University of Alberta, Edmonton, AB, Canada; 5 Division of Cardiology, Department of Medicine, University of Alberta, Edmonton, AB, Canada; 6 Department of Oncology, University of Alberta, Edmonton, AB, Canada; 7 Department of Biomedical Engineering, University of Alberta, Edmonton, AB, Canada; 8 KITE, Toronto Rehabilitation Institute, Toronto, ON, Canada

**Keywords:** Breast cancer, Time-restricted eating, Sedentary time, Healthy eating, CVD

## Abstract

Metabolic dysfunction and excess accumulation of adipose tissue are detrimental side effects from breast cancer treatment. Diet and physical activity are important treatments for metabolic abnormalities, yet patient compliance can be challenging during chemotherapy treatment. Time-restricted eating (TRE) is a feasible dietary pattern where eating is restricted to 8 h/d with water-only fasting for the remaining 16 h. The purpose of this study is to evaluate the effect of a multimodal intervention consisting of TRE, healthy eating, and reduced sedentary time during chemotherapy treatment for early-stage (I–III) breast cancer on accumulation of visceral fat (primary outcome), other fat deposition locations, metabolic syndrome and cardiovascular disease risk (secondary outcomes) compared with usual care. The study will be a two-site, two-arm, parallel-group superiority randomised control trial enrolling 130 women scheduled for chemotherapy for early-stage breast cancer. The intervention will be delivered by telephone, including 30–60-minute calls with a registered dietitian who will provide instructions on TRE, education and counselling on healthy eating, and goal setting for reducing sedentary time. The comparison group will receive usual cancer and supportive care including a single group-based nutrition class and healthy eating and physical activity guidelines. MRI, blood draws and assessment of blood pressure will be performed at baseline, after chemotherapy (primary end point), and 2-year follow-up. If our intervention is successful in attenuating the effect of chemotherapy on visceral fat accumulation and cardiometabolic dysfunction, it has the potential to reduce risk of cardiometabolic disease and related mortality among breast cancer survivors.

Breast cancer is the most commonly diagnosed malignancy in women worldwide, representing 25 % of annual new cancer cases^(1)^. In Canada, mortality for early-stage breast cancer has decreased by 48 % over the past 40 years, with a concurrent emergence of competing risks from non-cancer causes. For example, breast cancer survivors are at an increased risk for weight gain, obesity^(2)^, hypertension, type 2 diabetes^(3,4)^, heart failure and cardiovascular disease (CVD)-specific mortality^(5)^ compared with the general population.

The direct toxic effects of breast cancer treatment on the heart (cardiotoxicity) are well characterised as a contributor to elevated CVD risk^(2)^. However, metabolic dysfunction, characterised as insulin resistance, dyslipidemia and adipose tissue (AT) (fat) accumulation, is emerging as an important potential consequence of breast cancer treatment^(6)^. In non-cancer populations, metabolic syndrome, a cluster of three or more of dyslipidemia, hypertriacylglycerolaemia, abdominal obesity, hyperglycaemia and hypertension, is associated with a 2-fold increased risk of CVD^(7)^. An observational study found that chemotherapy treatment for breast cancer resulted in the new development of metabolic syndrome in 73 % of women^(8)^. Among all women in the observational study, there was a 108 % increase in insulin resistance and a 32 % average increase in inflammation during chemotherapy. In long-term follow-up, each criterion of metabolic syndrome was significantly worsened between end of chemotherapy and the 4–5-year follow-up period^(9)^. Evidence of various forms of metabolic dysfunction persisting at 5–30 years after chemotherapy treatment has also been reported^(10,11)^. Thus, metabolic toxicity from chemotherapy may represent an underappreciated contributor to the elevated CVD risk among breast cancer survivors.

Excess accumulation of fat within ectopic regions (e.g. visceral, intermuscular, or hepatic) triggers adverse metabolic effects and inflammation. In non-cancer populations, ectopic fat is more strongly linked to CVD risk than the total quantity, which is dominated by subcutaneous fat^(12)^. At the time of breast cancer diagnosis, the volume of visceral and intermuscular but not subcutaneous fat are linearly associated with CVD events within 6 years of follow-up, even among those with normal BMI and after adjustment for pre-existing CVD risk factors and treatment type^(13)^. Further, chemotherapy for breast cancer results in rapid (within 2–3 months) and persistent (≥1 year) accumulation of visceral and intermuscular fat^(14–16)^.

Diet and physical activity interventions play important roles in the treatment of metabolic abnormalities and body composition. Intermittent fasting is a pattern of eating involving voluntary abstinence from food alternated with periods of *ad libitum* eating (unrestricted amount and content). A variety of intermittent fasting formats have been examined during chemotherapy for breast cancer and have shown promise as safe interventions with positive effects on quality of life and treatment toxicity in women undergoing chemotherapy for breast cancer^(17)^. Time-restricted eating (TRE) is a form of intermittent fasting that involves *ad libitum* energy intake within a set window of time, commonly 8 h, followed by 16 h of fasting (16:8 TRE) daily. To our knowledge, TRE has not been studied during chemotherapy but shows promise as a feasible and effective therapy to mitigate metabolic toxicity from chemotherapy.

TRE has positive cardiometabolic health effects, including reduced body fat (whole body, visceral, liver and intermuscular^(18–22)^, inflammatory markers (IL-6 and TNF-*α*), adipokines (leptin and adiponectin), and improved lipid profile, fasting glucose, insulin sensitivity, and blood pressure^(18,19,23)^. TRE is also feasible, with adherence typically > 85 % among adults with obesity, metabolic syndrome or diabetes^(18)^. We recently demonstrated feasibility, safety and participant acceptability of 8 weeks of TRE in breast cancer survivors 3 years post-chemotherapy^(24)^. Our intervention also resulted in a 5 % reduction in visceral fat, a 1·0 kg decrease in whole-body fat mass, and a 15 % relative reduction in the Framingham 10-year CVD risk score^(24)^.

Appropriate nutrition and healthy eating practices can also reduce the burden of chemotherapy symptoms and improve health across the cancer continuum. Dietary intake and physical activity are inextricably linked to cardiometabolic disease risk through reciprocal contributions to energy balance and obesity. Outside of sleep, time spent not being physically active is considered sedentary time, defined as any waking behaviour done while sitting, reclining or lying down that expend ≤ 1·5 metabolic equivalents. Sedentary time has consistently shown to be higher in cancer survivors than controls^(25,26)^. Recently, we found that within the year after starting chemotherapy for breast cancer, whole-body and visceral fat accumulation were associated more strongly with an objectively measured increase in sedentary time (*r* = 0·47 & 0·51, respectively, both *P* < 0·001, unpublished) than either moderate-intensity physical activity or cardiorespiratory fitness, which are the focus of current exercise guidelines for cancer patients. These findings demonstrate a need for alternative approaches to energy balance during chemotherapy for early-stage breast cancer to attenuate fat accumulation.

The purpose of the Impact of Metabolic health Patterns And breast Cancer over Time in Women (IMPACT-Women) trial is to evaluate the effect of a multimodal behavioural intervention involving TRE, healthy eating and reduced sedentary time on ectopic AT and cardiometabolic profile *versus* usual cancer care in women receiving chemotherapy treatment for early-stage (I–III) breast cancer. We hypothesise that the intervention will attenuate ectopic AT, markers of metabolic dysfunction and CVD risk, and chemotherapy symptoms. Our secondary hypothesis is that the intervention effects on the cardiometabolic profile and incident diagnoses will persist to a 2-year follow-up.

## Methods

### Study design, randomisation and blinding

IMPACT-Women is a two-site, two-arm, parallel-group superiority randomised controlled trial. Participants will be randomised 1:1 to the two groups using permutated blocks with random block sizes of two and four, stratified by study site, chemotherapy type (adjuvant/neoadjuvant), and postmenopausal status (yes/no). Randomisation will occur after completion of baseline assessments using a blinded online electronic randomisation tool, with enrolment and assignment performed by a research coordinator. All outcome assessors will be blinded to group assignment. Blinding participants to a behavioural intervention is not possible but is not expected to bias our physiological outcomes. Participants’ treating oncologists will not be blinded as we expect that group assignment may need to be discussed during care.

### Ethics approval and registration

Both sites provide independent research ethics approval (HREBA.CC-22–0128; CAPCR.22–5374), which will be amended with protocol modifications. All participants will provide written informed consent. The trial is registered with clinicaltrials.gov (registration #NCT05432856).

### Participant eligibility

We will recruit adult females (≥ 18 years) diagnosed with early-stage (I–III) breast cancer who will receive any type of neoadjuvant (pre-surgical) or adjuvant intravenous chemotherapy. Individuals without access to a cellphone with Bluetooth capability (required for intervention adherence tracking) will be excluded. We will exclude individuals who have type 1 diabetes and type 2 diabetes if hemoglobin A1c > 10 % or are taking insulin or sulfonylureas. For safety reasons, we will also exclude participants with research MRI contraindications (e.g. pacemaker, pregnancy, and magnetic implants), a self-reported history of an eating disorder, BMI < 18·5 kg/m^2^ or clinical signs of cachexia (oncologist’s discretion) or who are otherwise lacking oncologist’s approval, ≥ 5 % weight loss within the last 6 months, and uncontrolled thyroid disorder. Individuals with a BMI > 40·0 kg/m^2^ will be excluded as a threshold to confirm body habitus fit within the MRI scanner bore. Those reporting currently working night/rotating shifts, eating within ≤10-hour window or consistently eating less than three meals per d in the past 3 months will be excluded because of the potential impact on the TRE intervention. Finally, we will exclude patients who cannot communicate in English or do not have medical clearance to perform maximal exercise testing.

### Participant recruitment

We anticipate that recruitment will start in January 2023 and be completed by December 2024. We will recruit from two Canadian university-affiliated cancer hospitals: the Princess Margaret Cancer Centre and the Cross Cancer Institute. At both sites, recruitment will be facilitated by the breast medical oncology circle of care who will request consent for contact by the research coordinator, who will screen and consent participants.

### Intervention

**Fig. 1.**
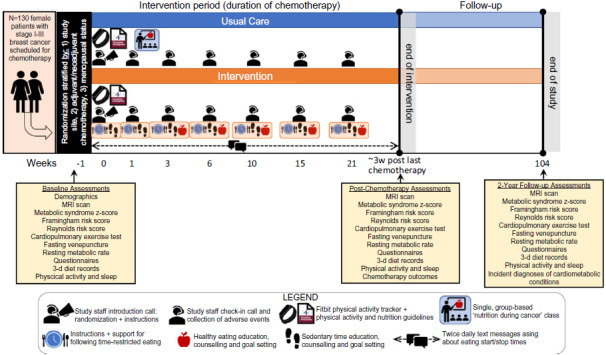
Intervention and follow-up diagram.

The intervention period will start shortly after randomisation and continue until the participant has completed all post-chemotherapy outcome assessments that occur 2–6 weeks after the last treatment (Fig. 1). The intervention is designed as a multimodal approach to improve metabolic health and will consist of:

#### Time-restricted eating

Participants will be asked to follow 16:8 TRE, but with flexibility incorporated to personalise the protocol to adapt to the dynamic nature of treatment symptoms, as well as their lifestyle, or preferences. TRE parameters can vary within the following pre-specified and evidence-based rules shown to improve cardiometabolic health: (1) adjustment of eating window length from 8 h up to 10 h; (2) self-selecting their eating window start time as long as end time is > 3 h before bedtime; (3) follow TRE as many days possible, but if time off is required, aim to limit it to 1–2 d off following at least five successive days each week.

#### Healthy eating

A registered dietitian will provide participants with a copy of Canada’s Food Guide, pre-determined nutritional education based on Canadian dietary guidelines, oncology guidelines for protein intake, individualised counselling and recommendations, and goal setting to promote healthy eating and overall nutritional well-being.

#### Sedentary time reduction

Both environmental and educational strategies will be incorporated to reduce sedentary time. Study staff will provide participants with the Canadian 24-h movement guidelines (which encompass recommendations for sedentary behaviour, physical activity and sleep), as well as education on sedentary time and the associated health impacts, counselling and goal setting on strategies to reduce sedentary time throughout the intervention period. All participants will receive a Fitbit physical activity tracker to enable tracking of daily step counts, reminders to move after periods of sedentary behaviour, and goal setting related to these activities. Goals will be reviewed and progressed throughout the intervention.

#### Intervention delivery

All components of the intervention will be delivered remotely by phone via evidence-based self-regulatory health behaviour support techniques^(27)^ including provision of education, ongoing tailored feedback, self-monitoring tools and goal setting. Participants will receive: (1) one-on-one 30–60-minute phone calls with a registered dietitian shortly after randomisation (week 0), and at 1, 3, 6, 10, 15, and 21 weeks and (2) daily self-monitoring via twice daily automated text messages about eating times and provision of a Fitbit physical activity tracker. At week 0, the registered dietitian will explain the rationale for TRE, including the pre-specified rules and provide instructions for starting TRE that evening. They will also review the participant’s baseline Fitbit data with them, provide initial education on sedentary time and introduce strategies to reduce sedentary time. Strategies to reduce sedentary time will include breaking up periods of sedentary time and a goal of increasing their daily step count between calls. At the follow-up support calls, feedback on TRE and sedentary time reduction adherence and counselling on barriers to adherence will occur. Potential symptoms and adverse events will be systematically tracked at these phone calls. The intervention focus on healthy eating and overall nutritional well-being will start in week 3 with an individualised assessment of dietary intake, education on Canada’s Food Guide and an individualised goal setting towards healthy eating. Since muscle loss is the primary nutrition problem that people with cancer experience, our nutrition intervention will focus on the protein intake recommendations from oncology nutrition guidelines^(28)^. In subsequent support calls, the registered dietitian will provide nutrition education, review success in achieving individualised nutrition goals, provide new goals as needed, and discuss barriers to behaviour change.

### Comparison group

The comparison group will receive the current standard of cancer and supportive care at our institutions including a single group-based ‘nutrition during cancer treatment’ class, a copy of Canada’s Food Guide, the Canadian 24-h movement guidelines and a Fitbit tracker. The control group will be instructed to maintain their usual dietary habits (timing, amount and type) outside of any recommendations given in the nutrition class for the duration of the intervention period. The research coordinator will perform the same number and timing of phone calls for participants in the comparison group as that in the intervention group to control for social support, to track the self-reported eating window to assess contamination and to collect adverse events.

### Outcomes

All outcomes will be measured at: (1) baseline (prior to first or second chemotherapy treatment), (2) 2–6 weeks post-last chemotherapy (typically lasting 12–18 weeks, depending on prescribed regimen) and (3) 2 years post-baseline. Participants who discontinue or deviate from intervention protocols will be asked to complete all follow-up assessments. The primary outcome is visceral AT volume at the end of chemotherapy. Secondary outcomes include the remaining AT pools (thigh intermuscular and intramuscular, liver, abdominal subcutaneous), metabolic syndrome z-score, 10-year Framingham and Reynolds risk scores, cardiorespiratory fitness, chemotherapy symptoms and quality of life. Homeostatic Model Assessment for Insulin Resistance (HOMA-IR), hemoglobin A1c, lipid profile, waist circumference, fasting glucose and blood pressure are tertiary outcomes as we expect they may only change among those who are metabolically unhealthy at baseline. Exploratory or potential mechanistic/explanatory outcomes will include leptin, adiponectin, IL-6, TNF-*α*, physical activity, dietary intake, resting metabolic rate, chemotherapy relative dose intensity and incident diagnoses of cardiometabolic conditions at the 2-year follow-up. Therefore, it is anticipated that data collection will be completed by September 2026. Safety will be evaluated using malnutrition guidelines^(29)^ and selected National Cancer Institute Common Terminology Criteria for Adverse Events (CTC-AE version 5) relevant to nutritional impact and muscle mass.

#### Visceral adipose tissue and regional adipose tissue pools

3T MRI (Siemens Prisma at both sites) will be used to quantify all AT volumes including the primary outcome of visceral AT. We will use standard chemical shift-encoding MRI and custom PROFIT1^(30)^ pulse sequences for image acquisition without contrast agents. Abdominal AT (both visceral and subcutaneous) and liver AT fraction will be captured from three axial slices prescribed at the centre of the third lumbar vertebra and the middle of liver, respectively. Thigh muscle and AT will be acquired from ten axial slices of both thighs starting 10 cm from the most distal portion of the femurs.

#### Metabolic syndrome severity

Metabolic syndrome will be classified according to the National Cholesterol Education Program Adult Treatment Panel III panel definition, and the severity z-score will be calculated through sex- and ethnic-specific equations^(31)^. The required blood samples will be collected by venipuncture following an overnight fast. Waist circumference and blood pressure will be measured by the study team using standardised procedures.

#### Cardiovascular risk estimate

The Framingham 10-year CVD risk score will be calculated using the Canadian Cardiovascular Society sex-specific scoring system incorporating points for age, total cholesterol, HDL, treated/untreated systolic blood pressure, diabetes, and smoking status. The Reynolds risk score will be calculated using the same variables as well as family history of heart attack (self-report) and C-reactive protein.

#### Cardiopulmonary exercise test and resting metabolic rate

We will measure cardiorespiratory fitness by the gold standard cardiorespiratory fitness assessment, an incremental-to-maximum exercise test with open-circuit indirect calorimetry. Monitoring before, during and after the test will include heart rate, pulse oximetry, blood pressure and the Borg rating of perceived exertion. Prior to the exercise test, resting metabolic rate will be assessed by using the same metabolic cart during 10–30 min of supine fasted rest.

#### Circulating biomarkers

Participants will be asked to complete an overnight water-only fast prior to venipuncture. Core laboratories at each site will analyse fasting insulin, glucose, lipid profile, hemoglobin A1c and C-reactive protein in fresh blood, while blood will be stored (in each location) at −80°C for later batch analysis of leptin, adiponectin, IL-6, and TNF-*α*, and potentially other related markers of cardiometabolic health that may help to uncover mechanisms.

#### Physical activity and sleep

A Fitbit wrist-worn tracker with heart rate monitoring will be used to collect data on average time spent performing physical activity of various intensity levels, sleep duration and quality data, and resting heart rate.

#### Dietary intake

Dietary intake will be assessed by 3-d diet records over two consecutive weekdays and one weekend day. Each of three 24-h records will be collected using the Canadian version of the Automated Self-Administered 24-h Dietary Assessment Tool (ASA24-Canada)^(32)^. Calorie, macronutrient and micronutrient intake will be analysed.

#### Questionnaires

Chemotherapy symptom presence and severity will be assessed by the Rotterdam Symptom Checklist, which has been validated in patients with cancer^(33)^. The RAND-36 Health Survey and the European Organisation for Research and Treatment of Cancer Quality of Life Questionnaire will be used to evaluate health-related quality of life. Menopausal symptom presence and severity will be assessed by the Menopause-specific Quality of Life questionnaire^(34)^. Fatigue will be assessed by the multidimensional fatigue inventory which is validated within cancer populations^(35)^. Presence and severity of depressive symptoms will be assessed using the Patient Health Questionnaire-9^(36)^. Psychosocial stress will be evaluated by the reliable and validated perceived stress scale^(37)^. Demographics, alcohol intake, smoking status and CVD risk factors will be collected by questionnaire. At the end of the intervention period in the intervention group only, acceptability of each component of the intervention will be assessed by the validated Diet Satisfaction Score tool^(38)^ and acceptability questions related to each aspect of the intervention based on a theoretical framework of healthcare intervention acceptability^(39)^.

#### Medical outcomes

We will access participant medical records to extract breast cancer diagnostic and treatment information and for chemotherapy outcomes (relative dose intensity, chemotherapy dose reductions and delays). We will also assess incident diagnoses of cardiometabolic conditions (hypertension, dyslipidemia, type 2 diabetes, CVD and non-alcoholic fatty liver disease) at the 2-year follow-up.

#### Safety outcomes

We will provide all participants with a Fibit body weight smartscale at baseline to take home to measure their weight first thing in the morning at least every 3 weeks. Based on global malnutrition guidelines^(29)^, if participants experience weight loss of 2 % or greater since their last weigh in, a registered dietitian will follow up with the participants to screen for the possible cause of weight loss and provide nutrition education and goals to minimise further weight loss. A 1-week follow-up will be scheduled to evaluate further weight changes. The participant’s treating oncologist will be notified if the participant continues to lose weight at the 1-week follow-up, or if weight loss at any 3-week weight check-ins exceeds 5%. Selected CTC-AE relevant to nutritional impact will be evaluated during the intervention at each of the support phone calls and through patient medical records collected via standard of care. At the support calls, participants will be asked if they have experienced each adverse event. If they respond positively, they will be further probed to ascertain the severity/grade and origin of the adverse event, and if relevant, the duration. Due to very low risk of major adverse events from our intervention, study investigators will assess safety outcomes on a case-by-case basis in lieu of a data safety monitoring committee.

### Sample size

The proposed sample size of *n=*130 was calculated using the primary outcome of visceral AT at the end of chemotherapy on GLIMMPSE software (https://glimmpse.samplesizeshop.org/). To calculate sample size, we used the average visceral AT change and its correlation between time points generated by our TRE pilot study (–4·8%) using identical MRI methods. For the comparison group, we used an average change of +6·6 % which we previously observed during 12 weeks of chemotherapy in a similar patient group at one of the included institutions^(40)^. For the time correlation matrix, we used the correlations between time points observed in our pilot data (*r* = 0·9866). However, to allow for an expected increase in variability induced by chemotherapy, we estimated a 25 % increase in variability (1·25 scale factor for covariance matrix) and standard deviation at the end of chemotherapy. Using the above values, *n=*116 total participants would provide 90 % power to detect a group × time interaction with *P* ≤ 0·05 for the Hotelling–Lawley trace test (which most closely approximates mixed models). We estimate a 12 % total loss to follow-up based on rates reported for TRE studies (∼10 %)^(41)^ plus 2 % for technical difficulties. Therefore, we will recruit *n*=130 total participants (*n*=65 per group).

### Analyses

We will evaluate for balance in baseline covariates between groups using *t* tests for continuous variables and Fisher’s exact test for categorical variables. Standardised differences between groups of 10 % or greater will be used as a threshold to signify clinically significant differences in baseline characteristics^(42)^. Our primary analyses will be a minimally adjusted intention-to-treat analysis wherein we will adjust for stratification factors (study site, adjuvant/neoadjuvant chemotherapy, and menopausal status)^(43)^. We will use generalised linear mixed models with a random effect for participant to account for individual variability. Time will be a repeated and fixed effect, with other fixed effects of group and group × time interaction. The distribution and link function that provides normally distributed residuals and/or the best model fit will be chosen for each outcome separately.

Planned secondary analyses include a per-protocol analysis (adherence to all aspects of intervention ≥ 5 of 7 d per week), a model with adjustment for covariates with clinically or statistically significant differences between groups at baseline, and a model with adjustment for covariates hypothesised to affect our outcomes including age and BMI.

### Data management

Data will be de-identified using a studyID and will be securely stored with access limited to study staff and investigators. Electronic data entry or extraction will be double-checked for accuracy.

## Discussion

Metabolic toxicity is an important emerging side effect from chemotherapy treatment resulting in rapid onset of metabolic dysfunction and persistent impairments after treatment completion^(8,10,11)^. This trial will examine the impact of a novel multimodal metabolic health intervention to attenuate changes to metabolic impairments including ectopic AT deposition and CVD risk. To our knowledge, no previous trials have implemented TRE or interventions to reduce sedentary time during active cancer treatment. Given that the causal independence of physical activity from diet is not well established, we chose to combine these two interventions which both show promise as feasible, safe and effective metabolic health interventions that could be easily scaled for implementation within clinical practice. The final component of our multimodal intervention focusses on improving healthy eating practices through nutritional education and counselling. Diet plays an obvious role in energy and protein intake and therefore energy and protein balance and ultimately cardiometabolic health^(44)^. A registered dietitian-led nutrition education and counselling intervention also provides an opportunity to aim to limit the spontaneous 20–30 % calorie restriction that typically occurs with *ad libitum* 16:8 TRE and to provide guidance on ensuring adequate protein intake in line with oncology guidelines^(28)^.

Our intervention is designed to address potential barriers to adherence common among patients receiving active cancer treatment. For example, we incorporated guidelines that allow participants to deviate from strict TRE in ways that are still supported by evidence for cardiometabolic health effects, including altering the length of TRE, selecting the start time of their eating window and guidance for taking a day off from TRE when required. Accounting for personal preferences in health interventions has been found to enhance recruitment and foster participant autonomy, enjoyment, and intervention adherence^(45,46)^. To overcome the common barrier of added study visits on top of extensive medical appointments, the intervention is delivered by phone, and in-person outcome assessments are only required three times over 2 years. This remote delivery format also increases potential for widespread implementation and future scalability to health systems and more remote communities. We will disseminate study results through academic conferences, peer-reviewed publications, and targeted patient and clinician events (e.g. survivorship groups and Grand Rounds).

A strength of this study is the use of 3T MRI as a non-invasive, safe (no ionising radiation), and highly reproducible technique to quantify the primary outcome (visceral AT), and other ectopic AT pools in the liver and thigh muscles. Our study will provide novel additions to the emerging evidence that rapid expansion of ectopic AT pools and their associated metabolic and/or cardiovascular dysfunction during breast cancer treatment plays a central role in the increased risk of CVD events among breast cancer survivors.

We have taken steps to minimise potential sources of bias. While blinding participants and their care team is not possible due to behavioural nature of the intervention and for patient safety, all outcome assessors will be blinded to the group assignment. In addition, participants in the comparison group will receive the same number and timing of calls to control for social impact and to assess contamination. One limitation is that we are using a parallel study design to implement a multimodal intervention which limits our ability to determine the individual effects of each component of the intervention.

In summary, the IMPACT-women study will evaluate the short- and longer-term effects of a multimodal metabolic health intervention during chemotherapy treatment for early-stage breast cancer on markers of metabolic health at the end of chemotherapy. The phone-based intervention consists of *ad libitum* TRE, healthy eating, and reducing sedentary time compared to usual cancer and supportive care. By attenuating the effect of chemotherapy on visceral AT accumulation and cardiometabolic dysfunction, our intervention has the potential to reduce risk of cardiometabolic disease and related mortality among breast cancer survivors.
